# Needs of social isolation, loneliness, and intergenerational interventions in the United States: a scoping review

**DOI:** 10.3389/fpubh.2024.1386651

**Published:** 2024-08-09

**Authors:** Jeremy Holloway, Dara L. James, Alyssa Robillard, Janice Hermer, Nanako Hawley, Owais Sayeed

**Affiliations:** ^1^Department of Geriatrics, School of Medicine and Health Sciences, University of North Dakota, Grand Forks, ND, United States; ^2^College of Nursing and Health Innovation, Arizona State University, Phoenix, AZ, United States; ^3^Library University Center, Arizona State University, Phoenix, AZ, United States; ^4^Department of Psychology, University of South Alabama, Mobile, AL, United States; ^5^School of Behavioral and Brain Sciences, University of Texas at Dallas, Richardson, TX, United States

**Keywords:** intergenerational programs, loneliness, social isolation, older adult, service-learning

## Abstract

Social isolation and loneliness are major health concerns for older adults in the United States. This scoping review examines the effectiveness of intergenerational interventions aimed at reducing social isolation and loneliness among older adults in the United States, specifically through programs that engage university students from healthcare-related fields in one-on-one settings with older adults, as reports of lacking geriatric training of healthcare students causes older adult neglect to persist in the healthcare workforce. The importance of addressing these issues is underscored by significant health risks and substantial economic burdens, with social isolation and loneliness potentially increasing mortality and costing Medicare an estimated $6.7 billion annually. Covering literature from 2010 to 2022, this review critically assesses the role of such interventions in fostering social connections and improving both physical and mental health outcomes. Despite the positive preliminary results indicating significant reductions in loneliness and enhancements in social networks among participants, the review highlights considerable gaps in current research, particularly in structured intervention curricula, demographic reporting and detailed intervention descriptions. This underscores the need for more rigorous and standardized research methodologies to better understand the effectiveness and potential of intergenerational programs as interventions against the detrimental effects of social isolation and loneliness among older adults.

## 1 Introduction

With an increase in the aging population, there is increasing concern regarding social isolation and loneliness among older adults within the United States (1). Emerging research shows that one out of three adults aged 45 or older feels lonely, while one out of four adults aged 65 or over is socially isolated (1–4). Recent articles have also shown loneliness increased among older adults after the COVID-19 pandemic (5). Social isolation is defined as a lack of social connections while loneliness is the development of feeling alone irrespective of the amount of social contact (6).

The adverse consequences of social isolation and loneliness among older adults extend beyond a mere reduction in their quality of life and significantly elevate the risk of mortality, with estimates ranging from 26 to 50% (5, 7, 8). This issue also carries a substantial economic burden, as addressing social isolation and loneliness in older adults has been estimated to cost Medicare a staggering $6.7 billion (9). Prior research has elucidated that older adults experiencing social isolation and loneliness are more likely to engage in detrimental behaviors such as increased tobacco and alcohol consumption (10, 11). Furthermore, the repercussions of loneliness and isolation encompass a range of physical ailments, including heart failure, diabetes, stroke, cognitive decline, and a higher incidence of suicide (12). It is crucial to emphasize that these impacts extend beyond physical health, encompassing mental health conditions such as dementia, depression, and delirium (13–16). Factors such as generational differences, bereavement, solitary living arrangements, and caregiving situations lacking a sense of purpose are found to contribute to the exacerbation of feelings of isolation and loneliness (11).

Studies show significant value of community-based service learning (CSL) as a crucial element of curriculum in medical and allied health education, particularly in the United States where the complexity of health care is ever-increasing. CSL programs, noted for their capacity to enrich student learning, civic responsibility, professionalism, and community sense, overcome traditional clinical placement limitations, facilitating interactions that enhance understanding of social determinants of health (17, 18). These programs, characterized by reciprocal academic-community partnerships, while scarcely studied for their community impact, address medical needs of under-resourced populations and are reported to enhance community experiences within medical education within Universities for University students in healthcare related fields (19–21).

In the context of an aging population, CSL serves as a strategic educational approach to prepare healthcare students for geriatric care, addressing gaps in service provision for underserved older adult populations and promoting interprofessional team learning (22–24). The project rationale highlights the increasing number of older adults, which necessitates a competent healthcare workforce for a demographic with growing chronic conditions and functional limitations (25, 26). Despite a projected increase in demand for geriatricians, there has been a decline in the number of these specialists, amplifying the need for primary care workforce training in geriatric care to prevent functional decline and reduce healthcare costs (26–29).

Accentuating the gap in Geriatrics training is the lack of inter-professional education and experiential Geriatrics related opportunities. At best, mock and real medical licensing exams show that most trainees exhibit average to below average knowledge of Geriatric principles. Finally, trainee attitudes about Geriatrics are suboptimal, suggesting the need to ramp up trainee engagement in Geriatrics. This educational framework not only enhances trainees’ knowledge and attitudes toward older adult care but also fosters essential communication skills within multidisciplinary teams, crucial for executing Comprehensive Geriatric Assessments that often reveal previously undetected health issues (30).

As older adults, due to social isolation and loneliness, become suspectable to aforementioned conditions, it is essential to examine the use of behavioral interventions, such as intergenerational programs, and their efficacy in reducing social isolation and loneliness among older adults. Intergenerational programs have garnered substantial attention in this context (31). Specifically, the implementation of one-on-one intergenerational programs that pair university students with older adults emerges as a promising avenue for addressing the multifaceted challenges of isolation and loneliness, concurrently offering students valuable service-learning experiences that can kindle an enhanced enthusiasm for geriatrics related experience (32). These programs have demonstrated their capacity to provide social connectivity, bolster positive self-perception, and cultivate self-assurance among older adults (32).

The efficacy of intergenerational programs, encompassing both pre-pandemic initiatives and those that have emerged during and after the pandemic, has not undergone comprehensive scrutiny within the existing scholarly discourse. This scoping review endeavors to provide an examination of all available academic articles within this domain, with its primary aim being the identification of prevailing gaps in the literature that necessitate further investigation through new primary research contributions. In the context of this systematic scoping review, particular attention is dedicated to the evaluation of one-on-one interventions, specifically those involving the pairing of university students in particular healthcare related fields with older adults. Furthermore, this article seeks to outline significant areas of needed progress within the existing body of literature concerning the alleviation of social isolation and loneliness through intergenerational interventions among the older adult population.

## 2 Materials and methods

### 2.1 Research question

This scoping review explores the existing literature pertaining to intergenerational interventions with university students in healthcare related fields for mitigating social isolation and loneliness among older adults in the United States. Our research question is: “What is the current state of research on intergenerational interventions involving college or university students in healthcare-related fields, aimed at reducing social isolation and loneliness among older adults in the United States, with a focus on studies conducted from the year 2010–2022?”

### 2.2 Inclusion and exclusion criteria

#### 2.2.1 Inclusion

This systematic scoping review considered intergenerational interventions conducted in the United States up to 2022. The scope further developed to focus on years 2010–2022 due to studies lack of applicable content prior to 2010. The study population comprised older adults aged 65 and above meeting with college or university students, particularly those pursuing degrees in healthcare-related fields. The primary focus of the interventions under consideration was to decrease isolation and loneliness in older adults, with an emphasis on social engagement. Furthermore, eligible studies involved interventions in a one-on-one format.

#### 2.2.2 Exclusion

This review excluded intergenerational parent–child dyads and studies involving elementary, junior high, and high school-aged participants. Studies conducted before the year 2010 were not included in the review. Additionally, studies with a primary focus other than decreasing social isolation and loneliness, such as those with a more educational orientation, were excluded. Research conducted outside of the United States was not considered. Finally, interventions delivered in a group setting or format were not within the scope of this review. The review also excluded abstracts, posters, and dissertations.

### 2.3 Search strategy

Our search strategy was implemented across multiple databases, including PubMed, PsychInfo, Embase, Academic Search Premier, and CINHAL Complete. The searches were carried out in June 2022, supplemented by a gray literature search in July 2022, with no date restrictions.

The main keywords/subjects were adjusted for each database for optimal searching. The core search string: “intergenerational relations” AND (elders OR elderly OR “older adults” OR seniors OR aged”) AND (loneliness OR “social participation” OR companionship OR connection OR “Social isolation” OR inclusion OR belonging OR engagement) AND (students AND (nursing OR medicine OR pharmacy OR “health occupations” OR “public health” OR college OR University OR “post secondary”)) All citations were uploaded into Covidence™ for de-duplication and screening.

**Figure 1:**
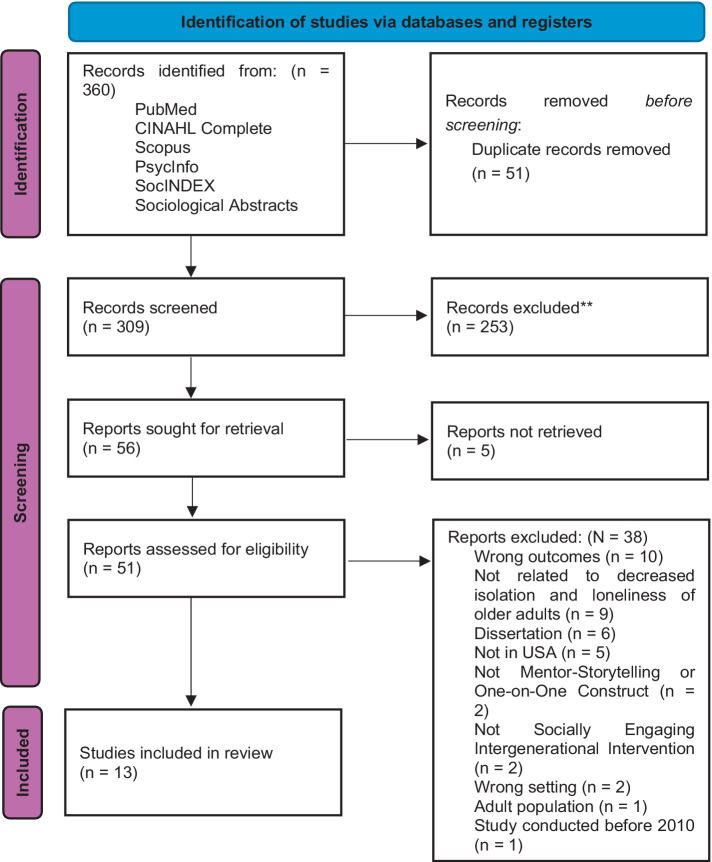
PRISMA process flow showcasing selection of articles.

The screening process comprised two stages: initial evaluation based on title and abstract, followed by a comprehensive assessment of full-text articles (see Figure 1 for details).

### 2.4 Data analysis and extraction

#### 2.4.1 Data extraction

The data extracted from the articles included the authors, year published, intergenerational interventions used, outcomes evaluated, scales of measurement for outcomes, type of study, and demographics of participants included in the articles.

#### 2.4.2 Data analysis

Data was summarized into Table 1 for convenience of comparison between the articles and due to the heterogeneity of results found within each article.

**Table 1:** Overview of selected articles used in the scoping review.

Ref.	Authors	Social Isolation/Loneliness Intervention	Outcomes evaluated	Scales of measurement used	Type of study	Was detailed demographic information included?
(36)	Harvey, Beck, Carr (2019)	IG Cognitive and language social media training	h, confidence	Montreal Cognitive Assessment, Cognitive Linguistic Quick Test, Quick Test of Cognitive Speed	Pre/post design	Age
(37)	Miller et al. (2022)	Pen pal IG program	Wellness, student awareness of social isolation & loneliness	5-point-likert scale, Fisher exact Test, Chi-squares test, Wilcoxon signed rank test	Pre/post design	Not available. Authors noted information not collected
(16)	Shenoi, Wong, Selleck (2022)	The Recreation and Education Network for Elder Wellness (RENEW)	Loneliness, social engagement	UCLA Loneliness Scale (revised) and pre/post surveys	Pre/post design	None
(33)	Zhang et al. (2022)	Virtual interprofessional Service-learning approach	Isolation, connectivity	Telehealth competency checklist	University course	None
(10)	Adepoju Et al. (2022)	Virtual field opportunity IG program	Loneliness, physical health, mental health	UCLA 3-item Loneliness Scale, CDC health related quality of life healthy days measure, Thematic Analysis	Mixed-Methods	Age, gender, race, location
(34)	Lee, Kim (2022)	Intergenerational Mentor-Up (IMU)	Feelings of social isolation and loneliness	eHealth literacy scale (eHEALS), 5-point likert scales, Cornwell, and Waite 9-item social isolation measure scale	Mixed-Methods	Age, gender, race
(38)	Juris et al. (2022)	Cyber-Seniors	Loneliness, social connectivity	Lubben Social Network Scale-6, UCLA 3-item loneliness scale	Pre/post pilot study	Age
(39)	Meuser et al. (2022)	Telecolloborative Service-Learning project (TSLP)	None	None	None	Older Adult Sexual Orientation, Student Majors
(40)	Weng (2019)	None	Feelings of social isolation and loneliness	Data analysis followed Creswell’s constant comparison strategy	Qualitative Case Study	Race, gender
(35)	Counts et al. (2022)	AHeAD (Aging Health and Development)	Student attitude, student interest	Perceptions of Aging and Elderly Inventory (PAEI), Elderly Patient Care Inventory (EPCI), SPSS software/open coding, and inferential statistical methods	Quasi Experimental	Student Gender & Race
(41)	Long & Knight (2022)	Cardinals CARE (Cardinals, Adopt, Residents, for Engagement)	None	None	Narrative Case Study	Race
(11)	Aguilera-Hernandez, Anderson, Negron (2019)	meetings, mealtimes, one-on-one leisure, and transportation	Social connection, loneliness, enjoyment	single likert-scale question, Anonymous surveys and semi-structured interviews were used to collect data	Mixed methods	Gender
(6)	Kylie Beausoleil, Jason Garbarino & Laura Foran Lewis (2022)	Aging is very personal (AVIP) service-learning program	Mood, engagement, social interaction, isolation, social interest	Questionnaire for Assessing the Impact of the COVID-19 Pandemic on Older Adults (QAICPOA), in-house survey created by the authors, questionnaire, lime survey, reflexive thematic analysis	Convergent parallel mixed-methods	Age, gender, employment, living situation

## 3 Results

### 3.1 Study characteristics

The information regarding characteristics on each of the studies, outlined in section 2.4.1, was extracted, and listed in Table 1. Demographic information varied, as did scales of measurement of the outcomes, which were primary areas of interest.

### 3.2 Demographics and study areas of interest

An area of interest for the researchers were the demographics. Out of the 13 articles included in the scoping review, approximately 5 of them did not provide any demographic information about the participants, making it challenging to ascertain the characteristics of the participants. Among the studies that did specify gender demographics, it was observed that female participants constituted a cumulative percentage of approximately 63.4%, while male participants accounted for approximately 36.6% across these studies.

Interestingly, none except 1 of the reviewed articles provided data on the sexual orientation of the participants, indicating a gap in this aspect of demographic information. One of the articles reported 7% of older adult participants identified as members of the LGBTQ+ community.

In terms of racial demographics, the cumulative summary of races involved across all the studies encompassed a range of non-Hispanic White participants making up around 48.5% of the total, non-Hispanic Black participants representing approximately 31.5%, Hispanic participants comprising about 4.7%, Asian-American participants constituting around 0.6%, and a category labeled as “Other” or “unclassified” including approximately 14.7% of the participants. These findings collectively underscore the need for future research to provide more comprehensive demographic data, particularly related to sexual orientation, to enhance the understanding of the diverse populations engaged in intergenerational interventions for reducing loneliness among older adults. Apart from Adepoju et al. (10), none related to the combination of social isolation and loneliness.

All but 1 article reported the number of student participants. The studies involved 15 graduate clinician participants from Speech-Language Pathology, focusing on social media training for cognitive and language interventions. Medicine students were notably included, with 74.4% of participants in one study coming from this discipline. Nursing students also participated, often alongside medical students and other healthcare fields.

Pharmacy students were part of intergenerational learning projects, contributing to the educational experience. Social Work was represented, with one program including 46 students, emphasizing the social aspects of healthcare. Students from Physician Assistant programs participated in discussions on interprofessional education. Audiology students took part in studies focused on virtual interprofessional learning. Gerontology students, particularly undergraduates, were involved in research aimed at reducing loneliness, highlighting the importance of age-related studies in healthcare education. Additionally, 35 students from Occupational Therapy were noted in various projects, and a student from Osteopathic Medicine also participated, showcasing the range of disciplines engaged in addressing the needs of older adults. From the available demographic data, all university participants were reported as enrolled in undergraduate, graduate, or professional programs related to caring professions (e.g., nursing, gerontology, social workers), with two articles that did not provide this information.

### 3.3 Intervention medium and duration

#### 3.3.1 Intervention medium

Of the 13 articles analysed, the vast majority provided insights into the mediums used for participant interactions. These communication channels encompassed a range of approaches, including purely in-person interactions in two articles, letter writing in one article, a combination of letter writing, emails, and phone calls in another article, a mix of phone calls and virtual video calls in four articles, a blend of in-person meetings and virtual video calls in one article, and exclusive reliance on virtual video calls in two articles. However, two articles did not include details about the interaction medium.

#### 3.3.2 Intervention duration

There was significant variation in the duration of the intervention programs, which did not have a common unit of measurement. This variation can be attributed to a variety of factors such as the length of academic courses and semesters, specific academic requirements, attrition rates and cost. The shorter articles that reported their duration ranged between a 4-week program conducted by Zhang et al. (33), and a total of 276 h within a single semester course by Lee and Kim (33, 34). The longest study, by Counts and colleagues 2019, reported the entire fall and spring semester of the 2018–2019 academic year (35).

### 3.4 Methods, scales of measurement, and loneliness outcomes

Four articles used a pre/posttest design, three mixed methods, one quasi-experimental, one qualitative, one narrative study, and two did not indicate a clear design. Across all articles, 12 different kinds of outcomes were measured (e.g., loneliness scores, isolation in older adults, student interest in working with older adults). Table 1 summarizes these outcomes.

Regarding older adult specific outcomes, only six articles specifically reported outcomes related to loneliness among older adults, but each study with this information reported significant reductions in older adult loneliness after participation in the intergenerational intervention. Specifically, after the older adults participated in a 6-week intervention that included committee meetings, mealtimes, 1:1 leisure, and transportation, Adepoju et al. (10) reported a decrease from 84.2 to 40% in UCLA Loneliness Scale scores.

Furthermore, Juris and colleagues (2022) found a 40.91% decrease in loneliness as measured by the Lubben Social Network Scale-6 and UCLA 3-item loneliness scale (38). This was supported by their qualitative findings that the program increased connectivity and prevented loneliness. Lastly, Adepoju et al. (10) found similar results utilizing a 3-item Loneliness Scale within a perceived social isolation measure. To strengthen these types of results, utilization of a randomized control group to compare results are plausible (10).

### 3.5 Conversational component

Interestingly, out of the 13 articles, 4 studies found that, within intergenerational activities, students and older adults predominantly found the conversation aspect to be the most enjoyable component, surpassing other activities. For example, one of the articles Zhang et al. (33) explains that while the programs involved various activities like committees, mealtime, one-on-one leisure, and transportation, the students consistently valued and relished engaging in meaningful dialogs with the older adults. This preference for conversations was evident across different activities, emphasizing the significance of the quality of interaction over specific tasks. For instance, committees allowed both generations to collaborate on shared goals, emphasizing the role of conversation in their engagement. Mealtime provided a conducive environment for comfortable social exchanges, leading to personal bonds. Even in one-on-one leisure activities, where the quantitative ratings suggested moderate enjoyment, qualitative data revealed that students highly valued the opportunities for connections and conversation. Thus, the students’ preference for conversations emerged as a notable and integral aspect of their participation in intergenerational programs, showcasing the significance of meaningful dialogs in fostering connections and mutual benefit between young and older adults.

## 4 Discussion

This scoping review explored the efficacy of intergenerational programs in mitigating social isolation and loneliness among older adults within the United States. Highlighting various interventions, the review illuminates the substantial benefits these programs offer to older adults and healthcare students in enhancing their educational experiences. While promising outcomes are reported, such as improved social connections and mood enhancements among older adults, the review also identifies significant gaps in the current literature, particularly in detailed curriculum descriptions and the long-term impacts of these interventions. This discussion specifically emphasizes the need for comprehensive future research to establish effective strategies and frameworks that can be consistently implemented across different settings.

Across the reviewed articles, several reported positive outcomes in addressing social isolation and loneliness among older adults. For instance, the study by Kylie Beausoleil, Jason Garbarino, and Laura Foran Lewis explored the impact of a virtual service-learning program during the COVID-19 pandemic and found that 86% of participants reported positive mood affecting changes post-program, with 71% reporting feelings of increased social connection from weekly sessions (6). Similar findings were found in supporting the healthcare student experience working with older adults (12). Such results reveal the usefulness of intergenerational interventions to decreasing social isolation and feelings of loneliness as a prevention in the lives of older adults while providing experiences that strengthen student experiences in healthcare related fields. Overall, future research should also aim to fill numerous gaps to provide a more widely established understanding of effective strategies for addressing social isolation and loneliness among older adults in the United States that include their perspectives on outcomes.

### 4.1 Older adult perspectives

Regarding the detailed exploration of older adult perspectives, among the 13 reviewed articles, only a few provided in-depth insights into the experiences and perspectives of older adults regarding social isolation, loneliness, and the effectiveness of interventions. While some studies shared valuable qualitative data on older adults’ experiences, many others primarily focused on outcomes and student perspectives. To better understand the nuances of social isolation and loneliness among older adults and to refine interventions, future research should prioritize conducting comprehensive qualitative inquiries, allowing older adults to express their thoughts, feelings, and preferences regarding these issues and the interventions designed to address them. Such an approach can provide a more holistic understanding and potentially lead to more tailored and effective interventions that resonate with the older adult population.

### 4.2 Student professions

In this scoping review focusing on the needs surrounding social isolation, loneliness, and intergenerational interventions in the United States, the studies included demonstrative engagements from a variety of healthcare professions. Notably, fields such as speech-language pathology, medicine, nursing, pharmacy, and social work were represented, indicating a broad interprofessional involvement. This diversity is crucial, as it reflects the complex and multifaceted approach required to tackle issues of social isolation and loneliness among older adults effectively.

However, the variability in professional representation and the depth of engagement across these studies also highlight a significant limitation: the potential inconsistency in training and outcomes. This scoping review underscores the necessity for future research to establish more standardized, interprofessional educational frameworks that can be uniformly implemented. Such frameworks would ensure that healthcare students across various disciplines receive consistent training on addressing social isolation and loneliness effectively.

Future studies should also aim to evaluate the long-term impacts of these intergenerational interventions on the quality of life for older adults and the professional development of healthcare students. This would help in understanding how effectively these educational interventions prepare students for future careers in geriatrics and contribute to sustained improvements in the lives of older adults.

### 4.3 Need for curriculum

The articles largely focus on the outcomes and benefits of intergenerational programs in alleviating loneliness among older adults and fostering connections with university students. While these studies have yielded promising results, a critical gap emerges in their limited descriptions of the curriculum and training methodologies employed within these interventions. This absence of detailed curriculum outlines and training protocols is of paramount concern for the academic and practical development of intergenerational programs. Shenoi and colleagues showed that, while this study is promising, the authors note that there is no set curriculum that would allow other researchers to reproduce these results (16). This absence of specific curriculum outlines and training protocols limits the ability to replicate and adapt these programs effectively in other settings, emphasizing the need for more comprehensive documentation of such crucial aspects in future research and development of intergenerational programs. A well-structured curriculum ensures consistency in program implementation, and aids in the systematic evaluation of its impact on both older adults and students (42). Moreover, explicit training guidelines are essential for program scalability and dissemination.

### 4.4 Building upon previous interventions

Apart from the adoption of general concepts of pen pals, letter writing, and phone calls, there appeared to be no intentional building upon previous interventions done in the United States, nor regard to intergenerational programs that may be occurring in the commercial industry for collaboration or desired insight. The lack of such connections among intergenerational programs, social isolation, and loneliness, reveal a gap in literature. Additionally, this supports the need for research to focus more on the experiences of the older adult and describe what the intervention’s methods and strategies used to decrease social isolation and/or loneliness for the older adult. Research should investigate and include post-intervention follow-up information that show long-term effects of participation. For example, the Intergenerational Getting AHeAD program shared that older adult residents participated in year-to-year as a way of interacting with younger adults (19). Having data to draw from across the years from participating in the program, would be useful with understanding how these issues can be addressed and if at all the program is effective. It is worth noting that this diversity in interaction methods can be partially attributed to the unique circumstances of studies conducted during the COVID-19 pandemic. Articles did not embellish on a previously established intervention, which may also explain the variety of the multiple mediums across articles.

A limitation to this scope review could be the key terms that were not used for the inclusion criteria. For example, one article aimed to evaluate social connectedness among older adults by assessing their experiences while participating in an intergenerational program (8). The term “social connectedness” is what one might aim for when addressing social isolation and loneliness among older adults. This limitation could have potentially excluded relative literature available on the topic.

Future research should also explore the efficacy of intergenerational programs in older adults living with HIV (categorized as 50 and above), a group for which social isolation and loneliness is elevated and often coupled with stigma. In the pursuit of evidence-based practice and replicability, it is imperative to provide a comprehensive understanding of the educational components and training strategies employed in these interventions.

## 5 Conclusion

This scoping review explores the landscape of intergenerational interventions aimed at addressing social isolation and loneliness among older adults in the United States. A total of 13 articles have been analyzed, shedding light on various approaches and outcomes of these programs.

The findings indicate that intergenerational interventions encompass a wide range of activities, from cognitive social media training to virtual discussion groups, technology mentoring, service learning, and more. While these interventions have demonstrated positive outcomes, it is essential to note that the extent to which they focus on reducing social isolation and loneliness varies. Some programs emphasize skill development, cognitive assessment, or academic goals, with social connectedness as a secondary outcome.

Demographics of both older adult and student participants vary across studies, with a predominantly older adult population aged 65 and above. Student participants often come from diverse fields of study in healthcare, such as medicine, nursing, social work, and gerontology, highlighting the multidisciplinary nature of these interventions. Several studies suggest positive impacts on reducing loneliness and increasing social engagement among older adults. Quantitative data, such as decreased loneliness scores, were reported in some cases, along with qualitative feedback supporting the positive outcomes. However, the depth of reporting on the experiences of older adults and the specific mechanisms through which these interventions address social isolation and loneliness varies across studies.

A critical gap is the lack of detailed descriptions of the curriculum and training methods used in these programs, making it challenging for researchers to replicate and adapt these interventions effectively. This absence of specific outlines and training protocols underscores the need for more comprehensive documentation in future research and development of intergenerational programs. Furthermore, there is a lack of intentional building upon previous interventions or collaboration with intergenerational programs in the commercial industry, highlighting a gap in the literature. Research should place more emphasis on older adults’ experiences and describe the methods and strategies used to address social isolation and loneliness. Long-term follow-up data could also provide valuable insights into the effectiveness of these programs. Additionally, the absence of certain specific key terms in the inclusion criteria are always considerable limitations, such as choosing to omit “social connectedness” among older adults among the key word search; However, this exclusion was purposeful to narrow the focus of the literature search to studies specifically aimed at mitigating the loneliness epidemic among older adults in the United States and preparing a generation of healthcare workers to serve this expanding demographic effectively.

Challenges and limitations identified include issues related to participant retention, scheduling conflicts, limited program duration, and a lack of standardized measures for assessing social isolation and loneliness. Furthermore, funding sources for these interventions are often not detailed, leaving questions about sustainability and scalability.

In conclusion, intergenerational interventions in the United States show promise in combating social isolation and loneliness among older adults, though there is room for improvement and standardization in program design, outcome assessment, and reporting. Future research should strive for a more comprehensive understanding of how these interventions impact the lives of older adults, with a particular focus on diverse demographics and the incorporation of standardized loneliness and social isolation measures. Additionally, exploring the long-term effects and scalability of these programs is crucial for addressing the growing concerns of social isolation and loneliness in aging populations.

## Author contributions

JeH: Writing – original draft, Writing – review & editing, Formal analysis. DJ: Writing – original draft, Writing – review & editing. AR: Writing – original draft, Writing – review & editing. JaH: Data curation, Methodology, Writing – original draft, Writing – review & editing. NH: Writing – original draft, Writing – review & editing. OS: Resources, Writing – original draft, Writing – review & editing.
